# Crystal structure of isobutyl 4-(2-chloro­phen­yl)-5-cyano-6-{(*E*)-[(di­methyl­amino)­methyl­idene]amino}-2-methyl-4*H*-pyran-3-carboxyl­ate

**DOI:** 10.1107/S2056989015000079

**Published:** 2015-01-10

**Authors:** T. Mohandas, C. Udhaya Kumar, S. Aruna Devi, B. Arul Prakasam, P. Sakthivel, T. Vidhyasagar

**Affiliations:** a Department of Physics, Shri Angalamman College of Engineering and Technology, Siruganoor, Tiruchirappalli, India; b Department of Chemistry, Annamalai University, Annamalainagar, Chidambaram, India; c Department of chemistry, Urumu Dhanalakshmi College, Tiruchirappalli, 620 019, India; d Department of Physics, Urumu Dhanalakshmi College, Tiruchirappalli, 620 019, India

**Keywords:** crystal structure, pyran derivative, C—H⋯O inter­actions

## Abstract

In the title compound, C_21_H_24_ClN_3_O_3_, the dihedral angle between the pyran ring (r.m.s. deviation = 0.037 Å) and the chloro­benzene ring is 88.56 (14)°. In the crystal, the mol­ecules are linked by C—H⋯O inter­actions, generating *C*(7) (001) chains.

## Related literature   

For the biological activities of pyran derivatives, see: Kitamura *et al.* (2006 ▸); Tangmouo *et al.* (2006 ▸); Cocco *et al.* (2003 ▸). For related structures, see: Park *et al.* (2012*a* ▸,*b* ▸).
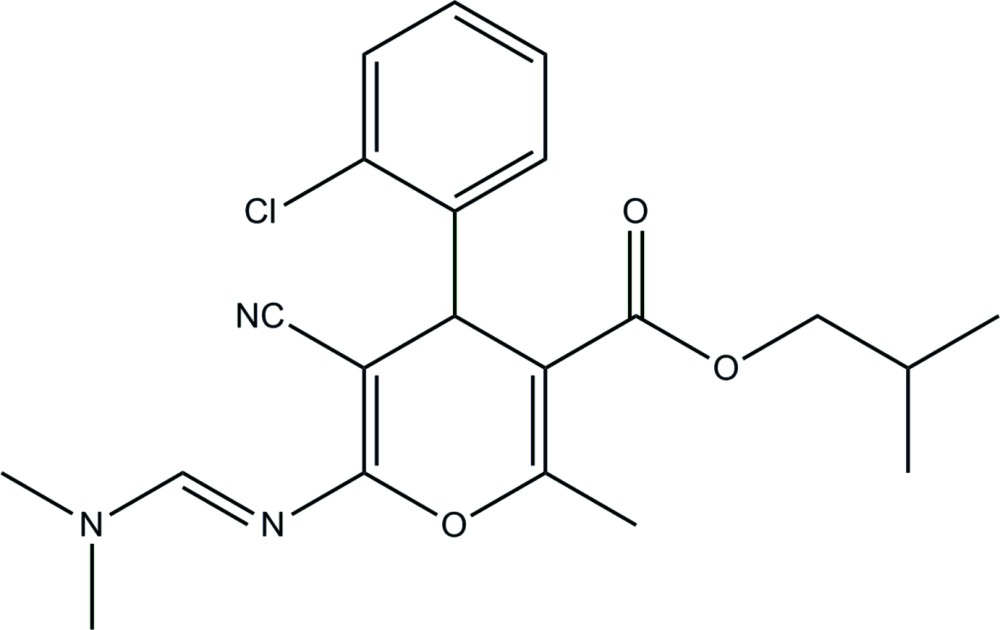


## Experimental   

### Crystal data  

C_21_H_24_ClN_3_O_3_
*M* *_r_* = 401.88
Monoclinic, 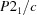

*a* = 15.6836 (16) Å
*b* = 15.2523 (13) Å
*c* = 9.3283 (8) Å
β = 105.016 (2)°
*V* = 2155.2 (3) Å^3^
*Z* = 4
Mo *K*α radiation
μ = 0.20 mm^−1^
*T* = 293 K
0.28 × 0.26 × 0.25 mm

### Data collection  

Bruker APEXII CCD diffractometer
Absorption correction: multi-scan (*SADABS*; Bruker, 2008 ▸) *T*_min_ = 0.985, *T*_max_ = 0.989
28118 measured reflections
5524 independent reflections
3062 reflections with *I* > 2σ(*I*)
*R*_int_ = 0.037

### Refinement  

*R*[*F* ^2^ > 2σ(*F* ^2^)] = 0.061
*wR*(*F* ^2^) = 0.217
*S* = 1.00
5524 reflections
258 parameters
H-atom parameters constrained
Δρ_max_ = 0.60 e Å^−3^
Δρ_min_ = −0.44 e Å^−3^

### 

Data collection: *APEX2* (Bruker, 2008 ▸); cell refinement: *SAINT* (Bruker, 2008 ▸); data reduction: *SAINT*; program(s) used to solve structure: *SHELXS97* (Sheldrick, 2008 ▸); program(s) used to refine structure: *SHELXL97* (Sheldrick, 2008 ▸); molecular graphics: *ORTEP-3 for Windows* (Farrugia, 2012 ▸) and *Mercury* (Macrae *et al.*, 2008 ▸); software used to prepare material for publication: *SHELXL97* and *PLATON* (Spek, 2009 ▸).

## Supplementary Material

Crystal structure: contains datablock(s) global, I. DOI: 10.1107/S2056989015000079/hb7341sup1.cif

Structure factors: contains datablock(s) I. DOI: 10.1107/S2056989015000079/hb7341Isup2.hkl

Supporting information file. DOI: 10.1107/S2056989015000079/hb7341Isup3.cml

**Fig. 1.**
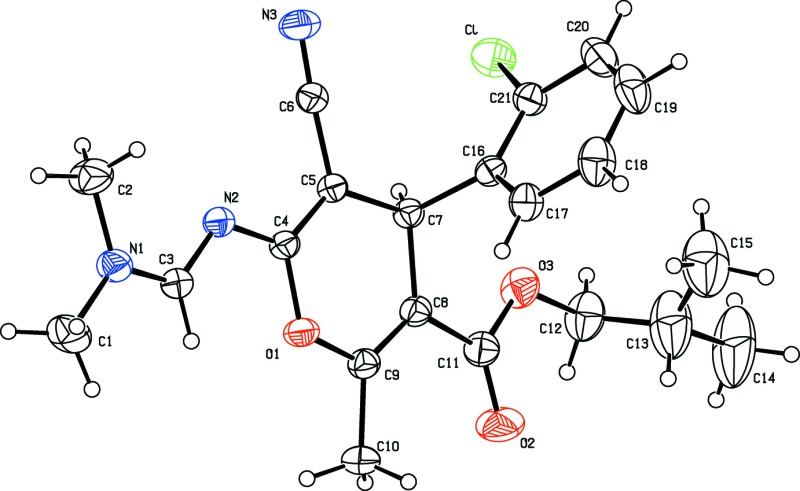
The molecular structure of the title compound with displacement ellipsoids drawn at 30% probability level.

**Fig. 2.**
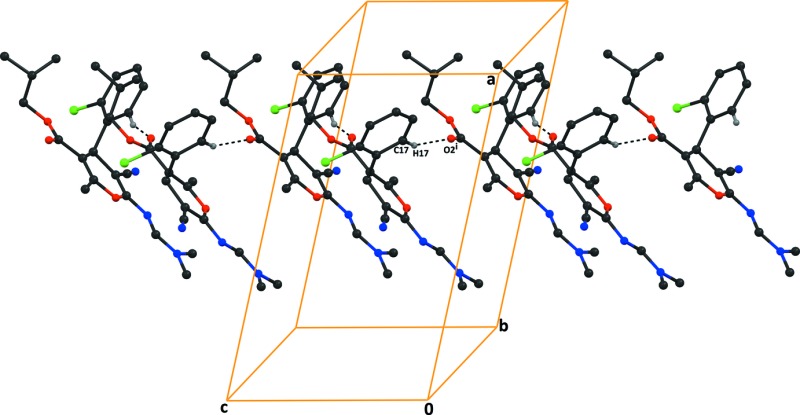
Part of crystal packing of the title compound showing the formation of C(7) chains running parallel to c axis.

. DOI: 10.1107/S2056989015000079/hb7341fig1.tif
The mol­ecular structure of the title compound with displacement ellipsoids drawn at 30% probability level.

c . DOI: 10.1107/S2056989015000079/hb7341fig2.tif
Part of crystal packing of the title compound showing the formation of C(7) chains running parallel to *c* axis.

CCDC reference: 1041846

Additional supporting information:  crystallographic information; 3D view; checkCIF report

## Figures and Tables

**Table 1:** Hydrogen-bond geometry (, )

*D*H*A*	*D*H	H*A*	*D* *A*	*D*H*A*
C17H17O2^i^	0.93	2.46	3.3368	157

Symmetry code: (i) 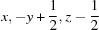.

## supplementary crystallographic information

### S1. Comment

2-Amino-4*H*-Pyran derivatives are an important class of heterocycles, which have considerable interest due to their useful biological properties including antimicrobial (Kitamura *et al.* 2006), antifungal (Tangmouo *et al.* 2006) and cancer therapy (Cocco *et al.* 2003).

The torsion angles of C8/C11/O3/C12 and N1/N2/C4/C5 are -178.75° and 177.08° respectively.

The bond distances and bond angles in the title compound agree very well with the corresponding values reported in closely related compound. (Park *et al.*2012(a,b)).

The crystal packing features C17—H17···O2 hydrogen bonds, which generate C(7)chains running parallel to the *c* axis.

### S2. Experimental

Isobutyl 6-amino-4-(2-chlorophenyl)-5-cyano-2-methyl-4*H*-pyran-3-carboxylate (1 mmol) and K_2_CO_3_ (1.2 equivalent) were stirred in dry DMF for 15 min in ice cold condition. Then, benzene-1,3,5-tricarbonyl trichloride (1 mmol) was added and stirred for an additional 25 min. After completion of the reaction, addition of water (50 ml), resulted the precipitate which was collected by filtration and washed with a large portion of cold water. The crude product thus collected was recrystallized from ethanol to yield colourless blocks.

### S3. Refinement

The positions of the hydrogen atoms bound to the O and C atoms are identified from the difference electron density maps and their distances are geometrically optimized. The H atoms associated with the hydroxyl groups are constrained to a distance of d(O—H) = 0.82 Å; and *U*iso(H) = 1.5*U*eq(O). The hydrogen atoms bound to the C atoms are treated as riding atoms, with d(C—H)=0.93 and *U*iso(H) = 1.2Ueq(C) for aromatic, d(C—H)=0.97 and *U*iso(H)=1.2Ueq(C) for methylene and d(C—H)=0.96 and *U*iso(H) =1.5*U*eq(C) for methyl groups.

### Crystal data

**Table:**

C_21_H_24_ClN_3_O_3_	*F*(000) = 848
*M**_r_* = 401.88	*D*_x_ = 1.239 Mg m^−^^3^
Monoclinic, *P*2_1_/*c*	Mo *K*α radiation, λ = 0.71073 Å
*a* = 15.6836 (16) Å	Cell parameters from 3064 reflections
*b* = 15.2523 (13) Å	θ = 1.3–29.7°
*c* = 9.3283 (8) Å	µ = 0.20 mm^−^^1^
β = 105.016 (2)°	*T* = 293 K
*V* = 2155.2 (3) Å^3^	Block, colourless
*Z* = 4	0.28 × 0.26 × 0.25 mm

### Data collection

**Table:**

Bruker APEXII CCD diffractometer	5524 independent reflections
Radiation source: fine-focus sealed tube	3062 reflections with *I* > 2σ(*I*)
Graphite monochromator	*R*_int_ = 0.037
ω & φ scans	θ_max_ = 29.7°, θ_min_ = 2.6°
Absorption correction: multi-scan (*SADABS*; Bruker, 2008)	*h* = −21→21
*T*_min_ = 0.985, *T*_max_ = 0.989	*k* = −20→20
28118 measured reflections	*l* = −12→12

### Refinement

**Table:**

Refinement on *F*^2^	Primary atom site location: structure-invariant direct methods
Least-squares matrix: full	Secondary atom site location: difference Fourier map
*R*[*F*^2^ > 2σ(*F*^2^)] = 0.061	Hydrogen site location: inferred from neighbouring sites
*wR*(*F*^2^) = 0.217	H-atom parameters constrained
*S* = 1.00	*w* = 1/[σ^2^(*F*_o_^2^) + (0.1039*P*)^2^ + 1.1568*P*] where *P* = (*F*_o_^2^ + 2*F*_c_^2^)/3
5524 reflections	(Δ/σ)_max_ = 0.033
258 parameters	Δρ_max_ = 0.60 e Å^−^^3^
0 restraints	Δρ_min_ = −0.44 e Å^−^^3^

### Special details

**Table:**

Geometry. All e.s.d.'s (except the e.s.d. in the dihedral angle between two l.s. planes) are estimated using the full covariance matrix. The cell e.s.d.'s are taken into account individually in the estimation of e.s.d.'s in distances, angles and torsion angles; correlations between e.s.d.'s in cell parameters are only used when they are defined by crystal symmetry. An approximate (isotropic) treatment of cell e.s.d.'s is used for estimating e.s.d.'s involving l.s. planes.
Refinement. Refinement of *F*^2^ against ALL reflections. The weighted *R*-factor *wR* and goodness of fit *S* are based on *F*^2^, conventional *R*-factors *R* are based on *F*, with *F* set to zero for negative *F*^2^. The threshold expression of *F*^2^ > σ(*F*^2^) is used only for calculating *R*-factors(gt) *etc*. and is not relevant to the choice of reflections for refinement. *R*-factors based on *F*^2^ are statistically about twice as large as those based on *F*, and *R*- factors based on ALL data will be even larger.

### Fractional atomic coordinates and isotropic or equivalent isotropic displacement parameters (Å^2^)

**Table:**

	*x*	*y*	*z*	*U*_iso_*/*U*_eq_	
C1	0.3162 (2)	0.1810 (2)	−0.0790 (4)	0.0762 (10)	
H1A	0.3371	0.2362	−0.0339	0.114*	
H1B	0.2546	0.1748	−0.0843	0.114*	
H1C	0.3247	0.1788	−0.1772	0.114*	
C2	0.3449 (2)	0.0213 (2)	−0.0425 (4)	0.0722 (10)	
H2A	0.3928	−0.0015	−0.0779	0.108*	
H2B	0.2918	0.0208	−0.1216	0.108*	
H2C	0.3370	−0.0145	0.0378	0.108*	
C3	0.42724 (17)	0.12629 (16)	0.1321 (3)	0.0441 (6)	
H3	0.4394	0.1840	0.1629	0.053*	
C4	0.53764 (16)	0.08439 (15)	0.3322 (3)	0.0380 (5)	
C5	0.58829 (15)	0.02517 (15)	0.4233 (3)	0.0381 (5)	
C6	0.57139 (17)	−0.06580 (16)	0.3917 (3)	0.0439 (6)	
C7	0.66429 (15)	0.04929 (15)	0.5534 (3)	0.0378 (5)	
H7	0.6549	0.0220	0.6432	0.045*	
C8	0.66744 (16)	0.14771 (16)	0.5739 (3)	0.0406 (6)	
C9	0.61304 (16)	0.20215 (16)	0.4809 (3)	0.0421 (6)	
C10	0.6059 (2)	0.29947 (17)	0.4866 (4)	0.0579 (8)	
H10A	0.6089	0.3174	0.5864	0.087*	
H10B	0.5506	0.3179	0.4222	0.087*	
H10C	0.6535	0.3258	0.4548	0.087*	
C11	0.73241 (19)	0.18383 (19)	0.7044 (3)	0.0515 (7)	
C12	0.8380 (2)	0.1463 (3)	0.9286 (3)	0.0742 (10)	
H12A	0.8175	0.1997	0.9652	0.089*	
H12B	0.8411	0.1013	1.0032	0.089*	
C13	0.9252 (3)	0.1612 (4)	0.9120 (5)	0.1227 (19)	
H13	0.9204	0.2177	0.8606	0.147*	
C14	0.9880 (3)	0.1800 (5)	1.0641 (6)	0.164 (3)	
H14A	0.9900	0.1300	1.1275	0.246*	
H14B	0.9674	0.2301	1.1074	0.246*	
H14C	1.0461	0.1915	1.0527	0.246*	
C15	0.9634 (3)	0.1055 (4)	0.8250 (6)	0.134 (2)	
H15A	0.9610	0.0459	0.8571	0.202*	
H15B	1.0238	0.1221	0.8361	0.202*	
H15C	0.9314	0.1105	0.7226	0.202*	
C16	0.74957 (16)	0.01276 (16)	0.5256 (3)	0.0393 (5)	
C17	0.78475 (18)	0.0516 (2)	0.4190 (3)	0.0512 (7)	
H17	0.7585	0.1019	0.3706	0.061*	
C18	0.8583 (2)	0.0169 (3)	0.3838 (4)	0.0713 (9)	
H18	0.8814	0.0440	0.3129	0.086*	
C19	0.8970 (2)	−0.0578 (3)	0.4536 (4)	0.0779 (11)	
H19	0.9457	−0.0817	0.4284	0.094*	
C20	0.8647 (2)	−0.0972 (2)	0.5595 (4)	0.0667 (9)	
H20	0.8911	−0.1477	0.6068	0.080*	
C21	0.79209 (18)	−0.06106 (17)	0.5962 (3)	0.0473 (6)	
N1	0.36500 (16)	0.11034 (14)	0.0093 (3)	0.0509 (6)	
N2	0.47213 (14)	0.06290 (13)	0.2112 (2)	0.0424 (5)	
N3	0.56164 (18)	−0.13952 (16)	0.3724 (3)	0.0672 (7)	
O1	0.55106 (12)	0.17257 (11)	0.3586 (2)	0.0477 (5)	
O2	0.74819 (18)	0.26007 (15)	0.7309 (3)	0.0899 (9)	
O3	0.77370 (14)	0.11992 (14)	0.7944 (2)	0.0611 (6)	
Cl	0.75622 (6)	−0.11215 (5)	0.73714 (9)	0.0711 (3)	

### Atomic displacement parameters (Å^2^)

**Table:**

	*U*^11^	*U*^22^	*U*^33^	*U*^12^	*U*^13^	*U*^23^
C1	0.081 (2)	0.0587 (19)	0.072 (2)	0.0163 (17)	−0.0101 (18)	0.0144 (16)
C2	0.082 (2)	0.0533 (18)	0.065 (2)	−0.0039 (16)	−0.0092 (17)	−0.0108 (15)
C3	0.0434 (14)	0.0369 (12)	0.0510 (15)	−0.0010 (11)	0.0101 (12)	0.0006 (11)
C4	0.0383 (12)	0.0324 (11)	0.0448 (13)	−0.0027 (10)	0.0137 (11)	−0.0002 (10)
C5	0.0380 (12)	0.0329 (12)	0.0443 (13)	−0.0017 (10)	0.0122 (10)	−0.0007 (10)
C6	0.0420 (13)	0.0377 (13)	0.0485 (14)	0.0014 (11)	0.0054 (11)	0.0003 (11)
C7	0.0397 (13)	0.0356 (12)	0.0376 (12)	−0.0014 (10)	0.0091 (10)	0.0011 (10)
C8	0.0416 (13)	0.0394 (13)	0.0425 (13)	−0.0029 (10)	0.0143 (11)	−0.0086 (11)
C9	0.0432 (13)	0.0375 (12)	0.0475 (14)	−0.0029 (10)	0.0155 (11)	−0.0062 (11)
C10	0.0623 (18)	0.0346 (13)	0.077 (2)	0.0009 (12)	0.0180 (16)	−0.0100 (13)
C11	0.0519 (16)	0.0524 (16)	0.0514 (15)	−0.0016 (13)	0.0155 (13)	−0.0158 (13)
C12	0.067 (2)	0.103 (3)	0.0459 (17)	−0.0102 (19)	0.0028 (15)	−0.0148 (18)
C13	0.073 (3)	0.203 (6)	0.083 (3)	−0.002 (3)	0.003 (2)	−0.047 (3)
C14	0.078 (3)	0.288 (9)	0.105 (4)	−0.023 (4)	−0.012 (3)	−0.078 (5)
C15	0.090 (3)	0.197 (6)	0.117 (4)	0.002 (4)	0.027 (3)	−0.057 (4)
C16	0.0407 (13)	0.0389 (12)	0.0366 (12)	−0.0036 (10)	0.0067 (10)	−0.0060 (10)
C17	0.0481 (15)	0.0588 (17)	0.0477 (15)	−0.0009 (13)	0.0142 (12)	0.0013 (13)
C18	0.0573 (19)	0.102 (3)	0.0607 (19)	−0.0026 (19)	0.0263 (16)	−0.0055 (19)
C19	0.0530 (19)	0.107 (3)	0.076 (2)	0.0185 (19)	0.0200 (17)	−0.019 (2)
C20	0.0556 (18)	0.068 (2)	0.070 (2)	0.0194 (15)	0.0043 (16)	−0.0088 (17)
C21	0.0467 (14)	0.0450 (14)	0.0462 (14)	0.0031 (11)	0.0049 (11)	−0.0036 (11)
N1	0.0532 (13)	0.0436 (12)	0.0497 (13)	0.0018 (10)	0.0022 (11)	0.0034 (10)
N2	0.0418 (11)	0.0361 (11)	0.0463 (12)	−0.0006 (9)	0.0059 (9)	0.0008 (9)
N3	0.0719 (17)	0.0366 (13)	0.0837 (19)	−0.0026 (12)	0.0030 (14)	−0.0057 (12)
O1	0.0537 (11)	0.0324 (9)	0.0525 (10)	−0.0020 (8)	0.0056 (8)	0.0006 (8)
O2	0.0990 (19)	0.0523 (13)	0.0964 (18)	−0.0006 (12)	−0.0142 (15)	−0.0325 (12)
O3	0.0618 (12)	0.0690 (14)	0.0450 (11)	−0.0063 (10)	0.0002 (9)	−0.0074 (10)
Cl	0.0811 (6)	0.0592 (5)	0.0707 (5)	0.0063 (4)	0.0155 (4)	0.0217 (4)

### Geometric parameters (Å, º)

**Table:**

C1—N1	1.448 (4)	C11—O2	1.201 (3)
C1—H1A	0.9600	C11—O3	1.339 (4)
C1—H1B	0.9600	C12—C13	1.433 (6)
C1—H1C	0.9600	C12—O3	1.447 (3)
C2—N1	1.449 (4)	C12—H12A	0.9700
C2—H2A	0.9600	C12—H12B	0.9700
C2—H2B	0.9600	C13—C15	1.411 (7)
C2—H2C	0.9600	C13—C14	1.531 (6)
C3—N2	1.306 (3)	C13—H13	0.9800
C3—N1	1.321 (3)	C14—H14A	0.9600
C3—H3	0.9300	C14—H14B	0.9600
C4—C5	1.349 (3)	C14—H14C	0.9600
C4—N2	1.355 (3)	C15—H15A	0.9600
C4—O1	1.374 (3)	C15—H15B	0.9600
C5—C6	1.429 (3)	C15—H15C	0.9600
C5—C7	1.510 (3)	C16—C21	1.385 (4)
C6—N3	1.143 (3)	C16—C17	1.389 (4)
C7—C8	1.513 (3)	C17—C18	1.385 (4)
C7—C16	1.532 (3)	C17—H17	0.9300
C7—H7	0.9800	C18—C19	1.373 (5)
C8—C9	1.337 (4)	C18—H18	0.9300
C8—C11	1.476 (4)	C19—C20	1.361 (5)
C9—O1	1.370 (3)	C19—H19	0.9300
C9—C10	1.491 (4)	C20—C21	1.385 (4)
C10—H10A	0.9600	C20—H20	0.9300
C10—H10B	0.9600	C21—Cl	1.742 (3)
C10—H10C	0.9600		
			
N1—C1—H1A	109.5	C13—C12—H12B	108.5
N1—C1—H1B	109.5	O3—C12—H12B	108.5
H1A—C1—H1B	109.5	H12A—C12—H12B	107.5
N1—C1—H1C	109.5	C15—C13—C12	121.7 (5)
H1A—C1—H1C	109.5	C15—C13—C14	112.2 (5)
H1B—C1—H1C	109.5	C12—C13—C14	109.4 (4)
N1—C2—H2A	109.5	C15—C13—H13	103.8
N1—C2—H2B	109.5	C12—C13—H13	103.8
H2A—C2—H2B	109.5	C14—C13—H13	103.8
N1—C2—H2C	109.5	C13—C14—H14A	109.5
H2A—C2—H2C	109.5	C13—C14—H14B	109.5
H2B—C2—H2C	109.5	H14A—C14—H14B	109.5
N2—C3—N1	121.5 (2)	C13—C14—H14C	109.5
N2—C3—H3	119.2	H14A—C14—H14C	109.5
N1—C3—H3	119.2	H14B—C14—H14C	109.5
C5—C4—N2	124.0 (2)	C13—C15—H15A	109.5
C5—C4—O1	120.3 (2)	C13—C15—H15B	109.5
N2—C4—O1	115.7 (2)	H15A—C15—H15B	109.5
C4—C5—C6	118.2 (2)	C13—C15—H15C	109.5
C4—C5—C7	123.8 (2)	H15A—C15—H15C	109.5
C6—C5—C7	117.9 (2)	H15B—C15—H15C	109.5
N3—C6—C5	176.4 (3)	C21—C16—C17	117.1 (2)
C5—C7—C8	109.67 (19)	C21—C16—C7	123.3 (2)
C5—C7—C16	108.51 (19)	C17—C16—C7	119.5 (2)
C8—C7—C16	112.41 (19)	C18—C17—C16	121.1 (3)
C5—C7—H7	108.7	C18—C17—H17	119.5
C8—C7—H7	108.7	C16—C17—H17	119.5
C16—C7—H7	108.7	C19—C18—C17	119.9 (3)
C9—C8—C11	119.4 (2)	C19—C18—H18	120.1
C9—C8—C7	122.7 (2)	C17—C18—H18	120.1
C11—C8—C7	117.9 (2)	C20—C19—C18	120.5 (3)
C8—C9—O1	122.2 (2)	C20—C19—H19	119.7
C8—C9—C10	129.5 (2)	C18—C19—H19	119.7
O1—C9—C10	108.3 (2)	C19—C20—C21	119.2 (3)
C9—C10—H10A	109.5	C19—C20—H20	120.4
C9—C10—H10B	109.5	C21—C20—H20	120.4
H10A—C10—H10B	109.5	C20—C21—C16	122.1 (3)
C9—C10—H10C	109.5	C20—C21—Cl	117.1 (2)
H10A—C10—H10C	109.5	C16—C21—Cl	120.8 (2)
H10B—C10—H10C	109.5	C3—N1—C1	121.2 (2)
O2—C11—O3	122.4 (3)	C3—N1—C2	120.6 (2)
O2—C11—C8	126.3 (3)	C1—N1—C2	118.1 (2)
O3—C11—C8	111.3 (2)	C3—N2—C4	118.2 (2)
C13—C12—O3	114.9 (3)	C9—O1—C4	120.97 (19)
C13—C12—H12A	108.5	C11—O3—C12	117.1 (3)
O3—C12—H12A	108.5		
			
N2—C4—C5—C6	−0.7 (4)	C5—C7—C16—C17	72.4 (3)
O1—C4—C5—C6	179.6 (2)	C8—C7—C16—C17	−49.1 (3)
N2—C4—C5—C7	176.7 (2)	C21—C16—C17—C18	1.0 (4)
O1—C4—C5—C7	−3.0 (4)	C7—C16—C17—C18	−175.6 (3)
C4—C5—C6—N3	175 (5)	C16—C17—C18—C19	0.7 (5)
C7—C5—C6—N3	−3 (5)	C17—C18—C19—C20	−1.2 (5)
C4—C5—C7—C8	6.1 (3)	C18—C19—C20—C21	0.0 (5)
C6—C5—C7—C8	−176.4 (2)	C19—C20—C21—C16	1.8 (5)
C4—C5—C7—C16	−117.0 (3)	C19—C20—C21—Cl	−177.8 (3)
C6—C5—C7—C16	60.5 (3)	C17—C16—C21—C20	−2.3 (4)
C5—C7—C8—C9	−4.2 (3)	C7—C16—C21—C20	174.2 (2)
C16—C7—C8—C9	116.6 (3)	C17—C16—C21—Cl	177.3 (2)
C5—C7—C8—C11	174.5 (2)	C7—C16—C21—Cl	−6.3 (3)
C16—C7—C8—C11	−64.7 (3)	N2—C3—N1—C1	−179.2 (3)
C11—C8—C9—O1	−179.6 (2)	N2—C3—N1—C2	−0.5 (4)
C7—C8—C9—O1	−0.9 (4)	N1—C3—N2—C4	177.1 (2)
C11—C8—C9—C10	−0.3 (4)	C5—C4—N2—C3	179.5 (2)
C7—C8—C9—C10	178.4 (3)	O1—C4—N2—C3	−0.8 (3)
C9—C8—C11—O2	−7.4 (5)	C8—C9—O1—C4	5.0 (4)
C7—C8—C11—O2	173.9 (3)	C10—C9—O1—C4	−174.5 (2)
C9—C8—C11—O3	172.5 (2)	C5—C4—O1—C9	−3.0 (3)
C7—C8—C11—O3	−6.3 (3)	N2—C4—O1—C9	177.3 (2)
O3—C12—C13—C15	−41.0 (7)	O2—C11—O3—C12	1.1 (4)
O3—C12—C13—C14	−174.6 (5)	C8—C11—O3—C12	−178.7 (2)
C5—C7—C16—C21	−104.0 (3)	C13—C12—O3—C11	−86.1 (5)
C8—C7—C16—C21	134.5 (2)		

### Hydrogen-bond geometry (Å, º)

**Table:**

*D*—H···*A*	*D*—H	H···*A*	*D*···*A*	*D*—H···*A*
C17—H17···O2^i^	0.93	2.46	3.3368	157

Symmetry code: (i) *x*, −*y*+1/2, *z*−1/2.
