# Supplementary figures and images for: Crystal structure of isobutyl 4-(2-chloro­phen­yl)-5-cyano-6-{(E)-[(di­methyl­amino)­methyl­idene]amino}-2-methyl-4H-pyran-3-carboxyl­ate
Source: Acta Crystallogr E Crystallogr Commun. 2015 Jan 10;71(Pt 2):o101–2. doi: 10.1107/S2056989015000079 (PMC4384599; doi:10.1107/S2056989015000079)

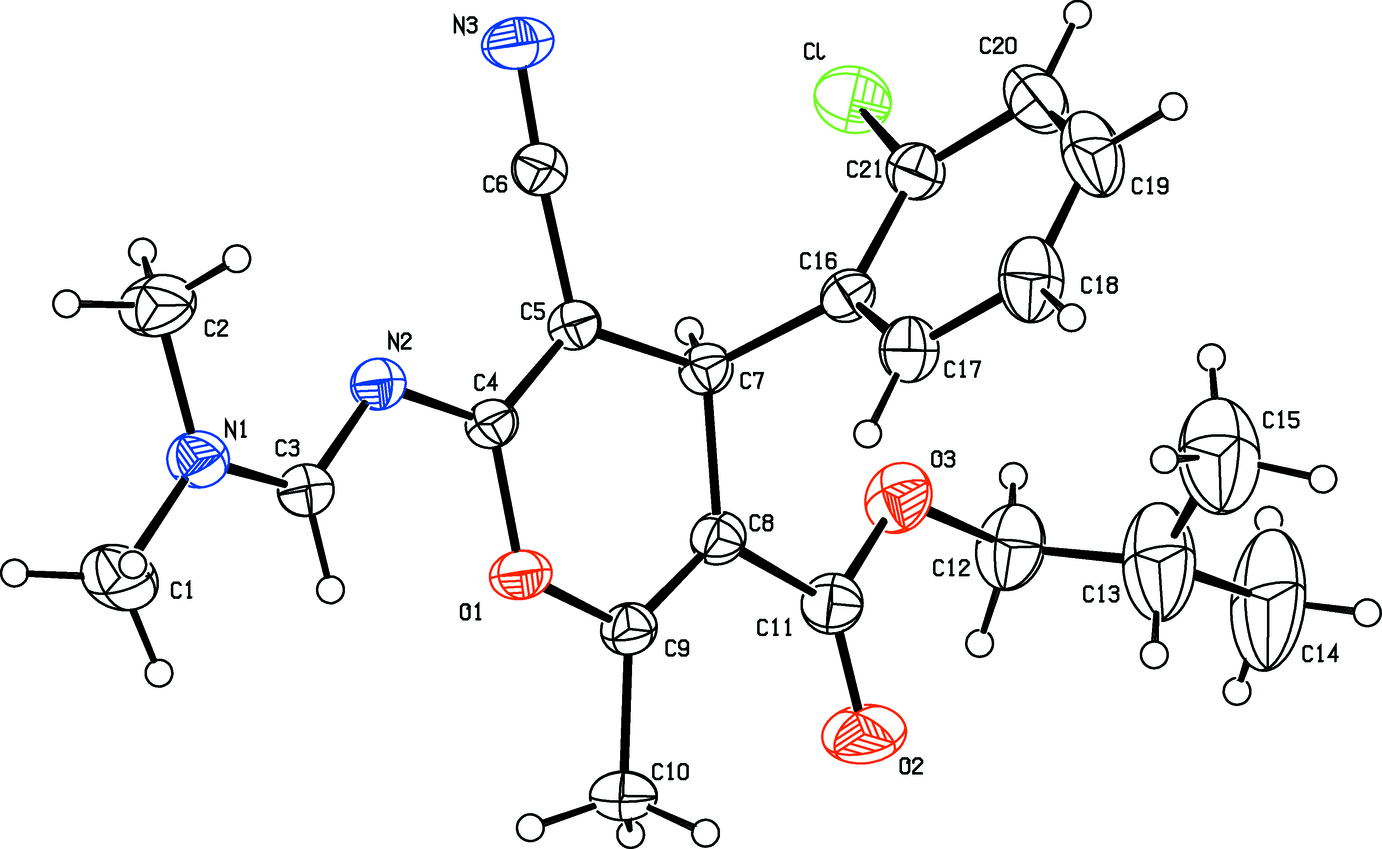

Supplement: Supplementary file 4 [file e-71-0o101-fig1.tif]

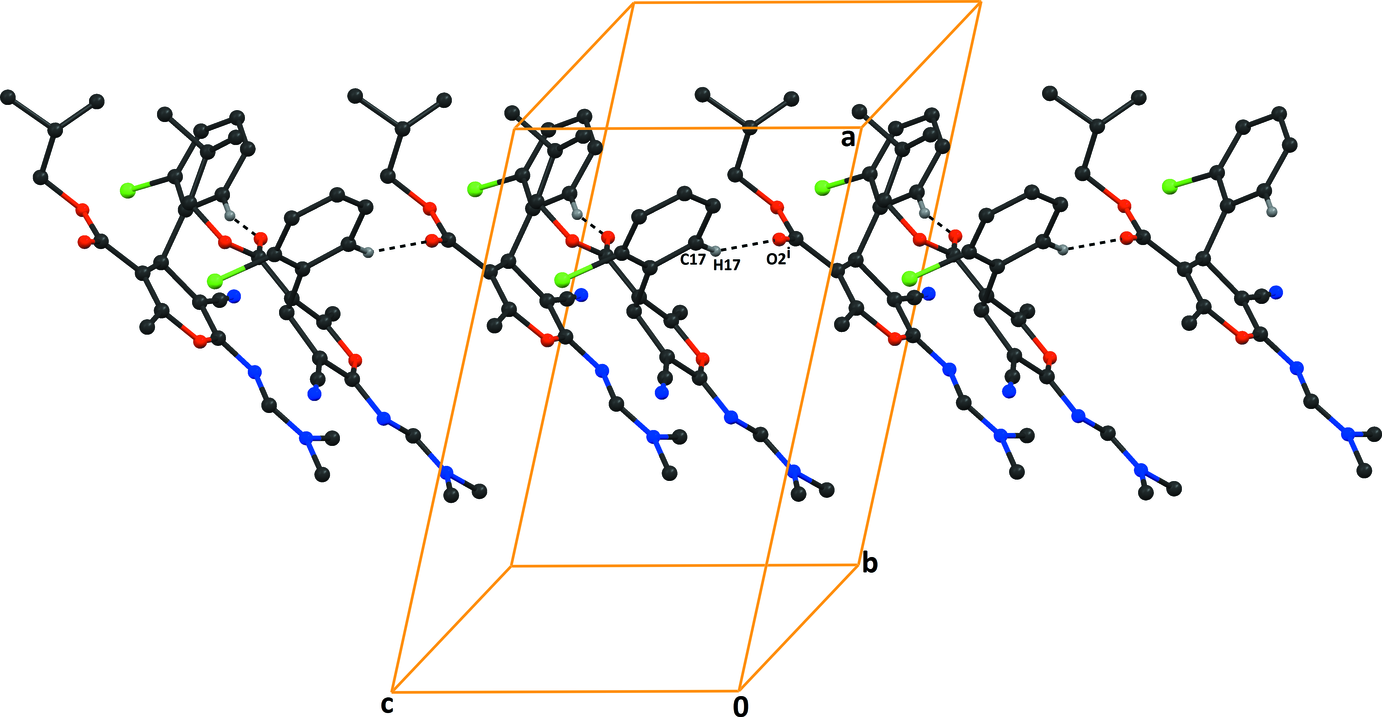

Supplement: Supplementary file 5 [file e-71-0o101-fig2.tif]
